# Headache following vaccination against COVID-19 among healthcare workers with a history of COVID-19 infection: a cross-sectional study in Iran with a meta-analytic review of the literature

**DOI:** 10.1186/s13005-023-00363-4

**Published:** 2023-05-19

**Authors:** Somayeh Nasergivehchi, Mansoureh Togha, Elham Jafari, Mehrdad Sheikhvatan, Donya Shahamati

**Affiliations:** 1 Department of Headache, Iranian Center of Neurological Research, Neuroscience Institute, Tehran University of Medical Sciences, Tehran, Iran; 2 Department of Neurology, Baharloo University Hospital, Tehran University of Medical Sciences, Tehran, Iran; 3 Department of Headache, Neurology Ward, School of Medicine, Sina University Hospital, Tehran University of Medical Sciences, Tehran, Iran; 4 Tehran University of Medical Sciences, Tehran, Iran; 5 Heidelberg University Hospital, Heidelberg, Germany; 6 Faculty of Nutrition Sciences and Food Technology, National Nutrition and Food Technology Research Institute, Shahid Behehshti University of Medical Sciences Tehran, Tehran, Iran

**Keywords:** COVID-19, Vaccination, Post-vaccination headache

## Abstract

**Background:**

There is evidence of the occurrence of headache after vaccination against COVID-19. However, only a few studies have examined the headache characteristics and related determinants, especially among healthcare workers with a history of COVID-19 infection.

**Methods:**

We evaluated the incidence of headaches after injection of different types of COVID-19 vaccine to determine factors relating to the incidence of headache after vaccination among the Iranian healthcare workers who had previously contracted COVID-19. A group of 334 healthcare workers with a history of COVID-19 infection were included and vaccinated (at least one month after recovery without any COVID-19 related symptoms) with different COVID-19 vaccines. The baseline information, headache characteristics and vaccine specifications were recorded.

**Results:**

Overall, 39.2% reported experiencing a post-vaccination headache. Of those with a previous history of headache, 51.1% reported migraine-type, 27.4% tension-type and 21.5% other types. The mean time between vaccination and headache appearance was 26.78 ± 6.93 h, with the headache appearing less than 24 h after vaccination in most patients (83.2%). The headaches reached its peak within 8.62 ± 2.41 h. Most patients reported a compression-type headache. The prevalence of post-vaccination headaches was significantly different according to the type of vaccine used. The highest rates were reported for AstraZeneca, followed by Sputnik V. In regression analysis, the vaccine brand, female gender and initial COVID-19 severity were the main determinants for predicting post-vaccination headache.

**Conclusion:**

Participants commonly experienced a headache following vaccination against COVID-19. Our study results indicated that this was slightly more common in females and in those with a history of severe COVID-19 infection.

## Introduction

With the onset of the COVID-19 pandemic in December 2019, efforts began to provide effective and safe drugs to treat the disease and prevent its development. In line with what has been observed in previous pandemics, the efforts to produce effective vaccines increased rapidly. A few months after the start of such efforts and with the rapid spread of COVID-19 and its variants, the first generations of effective immunogenic vaccines were introduced and gradually approved by international scientific reference committees, including the Centers for Disease Control and Prevention (CDC) and World Health Organization (WHO) [1, 2].

It did not take long to generate various brands of vaccines in countries such as the United States, China, Germany, Japan, India, Russia, and even in developing countries such as Iran and Cuba, and introduce them to the world [3–5]. However, as the production and commercialization of these vaccines accelerated, concerns arose. First, with the emergence of COVID-19 variants, especially the Delta strain, concern increased about reduced immunogenicity against the virus variants. The protective effects of some vaccines initially having an immunogenicity level of over 90% were seen to decrease to less than 70% [6, 7]. More importantly, following inoculation with various vaccine brands against COVID-19, potential and, rarely, life-threatening side effects were reported. Common side effects for the COVID-19-vaccines included local inflammation, headache, muscle pain, nausea, fatigue, fever and chills [8]. Anaphylaxis, thromboembolic events, myocarditis, pericarditis and even death were rarely reported, but seriously called into question the safety of some brands [9].

One of the most common side effects following injection of the different brands of COVID-19 vaccine has been headache. According to the Zoe Health Study, the overall prevalence of headache after vaccination by Pfizer-BioNTech vaccine ranged from 25 to 42% [10]. The CDC Trusted Source page reported that approximately one-third of people experienced severe post-vaccination headache, regardless of the type or brand of vaccine, at a rate of 1% after the first dose and 3% after the second dose [11].

One study in Italy reported on the increased likelihood of headache after vaccination by AstraZeneca vaccine, followed by the Pfizer vaccine [12]. Ekizoglu et al. assessed the history of headache following influenza vaccination and during Covid-19. They found that 30.6% healthcare personnel had experienced headache following Covid vaccination that was more common in females with pre-existing primary headaches, thyroid disorders, headache during COVID-19, or headache related to the influenza vaccine [15]. Sekiguchi et al. in their study in Japan performed a survey on nursing staff. Their result demonstrated that participants with the history of headache (migraineurs and non-migraineurs) will develop more headache compare to the healthy controls [13].

However, overall information about headache following those brands, as well as others, has been limited and requires further evaluation. This is especially important for individuals who have experienced COVID-19 infection prior to vaccination. This study evaluated and compared the incidence of headache following inoculation with different types of commonly used COVID-19 vaccines and determined the factors related to the incidence of headache following vaccination among selected Iranian healthcare workers who had previously recovered from COVID-19.

## Materials and methods

The participants in this cross-sectional study comprised 334 healthcare personnel who had initially recovered from COVID-19 infections of different intensities. According to our institutional protocol, these individuals were vaccinated at least one month after recovery with one of the brands of COVID-19 vaccine mentioned above between April and September 2021. The brands of corona vaccine that were commonly used among healthcare workers in this study included AstraZeneca, Sinopharm (China), Sputnik V (Russia), Bharat (India) and COVIran Barekat (Iran).

An online questionnaire designed to cover all the necessary data. This questionnaire included the demographics characteristics, the brand of vaccine administered, severity of initial COVID-19 infection (defined as quarantine at home, hospitalization in an isolated ward or ICU), concomitant clinical symptoms, PCR positivity after vaccination, rate of analgesic use after vaccination and COVID-19 positivity between two vaccine doses. In addition, information related to the post-vaccination headache, including time duration between vaccination and headache occurrence, time to reaching peak intensity after onset, pattern and location of the headache and medication used for headache relief also were assessed.

All patients were reassured about the privacy of their information and, after explaining the objectives of the project, verbal consent was obtained from all. The study endpoint was to determine the prevalence of headache and its characteristics following the use of each vaccine brand and then to determine the effect of vaccination of the different brands while adjusting for gender, initial COVID-19 severity and previous history of headache. In this regard, the severity of the COVID-19 was determined based on the Criteria for Clinical Severity of Confirmed COVID-19 as released by WHO [1, 2].

For statistical analysis, results were presented as mean ± standard deviation (SD) for the quantitative variables and were summarized by frequency (percentage) for categorical variables. Continuous variables were compared using the *t*-test or Mann-Whitney test whenever the data did not appear to have a normal distribution or when the assumption of equal variance was violated across the study groups. The multivariable logistic regression model was employed to examine the effect of type of vaccine on post-vaccination headache as adjusted for gender, history of headache and COVID-19 severity. P-values of ≤ 0.05 were considered statistically significant. The statistical software SPSS (version 23.0) for Windows (IBM; USA) was used for statistical analysis.

## Results

A total of 334 hospital staff members who had a history of COVID-19 infection and had subsequently been vaccinated with different brands of vaccines in Iran were assessed (Table 1). The average age of participants was 36.62 ± 4.36 year. of which 72.8% were female and 27.2% were male. Of the vaccines used, 12.9% were vaccinated with AstraZeneca, 16.2% with Sinopharm, 62.3% with Sputnik V, 6.9% with Bharat and 1.8% with other brands. The initial COVID-19 severity of the participants was assessed and it was determined that 62.6% had quarantined at home, 32.3% had been hospitalized in a general ward and 5.1% had been admitted to an ICU.

**Table 1:** Baseline characteristics of study population (n = 334)

Gender (%)	Male	91 (27.2)
	Female	243 (72.8)
Type of vaccine (%)	Astrazeneca	43 (12.9)
	Sinopharm	54 (16.2)
	Sputnik	208 (62.3)
	Baharat	23 (6.9)
	Others	6 (1.8)
COVID-19 severity (%)	Hospitalization	108 (32.3)
	ICU admission	17 (5.1)
	Quarantine at home	209 (62.6)
Concomitant symptoms (%)	Joint pain	59 (17.7)
	Chilling	13 (3.9)
	Muscular pain	72 (21.6)
	Runny nose	23 (6.9)
	Sleep problem	6 (1.8)
	Dizziness	8 (2.4)
	Neural symptoms	20 (6.0)
PCR positivity after vaccination (%)		2 (0.6)
Analgesic use after vaccination (%)		28 (8.4)

Overall, 39.2% of participants reported experiencing post-vaccination headache and 30.8% of participants reported a history of headache. Of those, 51.1% characterized their previous headaches as of the migraine type, 27.4% as tension type and 21.5% as other types. The mean time between injection of the vaccine and the onset of headache was 26.78 ± 6.93 h. Most participants (83.2%) reported the onset of headache to be less than 24 h after vaccination. They reported the headache reaching a peak within about 8.62 ± 2.41 h after the onset and the overall duration of the headache to be 4.22 ± 1.26 h. In 50% of participants, the headache duration was less than 6 h.

With respect to the symptoms accompanying the post-vaccination headache, the most frequent was nausea (9.2%), followed by sensitivity to noise (6.9%) and photophobia (4.6%). In most patients, the headache was of the compression type and most reported that the headache was felt diffusely in various parts of the head. The severity of headache in most participants (93.0%) was such that they resorted to the use of some type of analgesic (Table 2).

**Table 2:** Characteristics of headache after vaccination in study population (n = 334)

Prevalence of post-vaccination headache (%)		131 (39.2)
Previous history of headache (%)	Migraine	67 (51.1)
	Tension	36 (27.4)
	Other types	28 (21.5)
Mean time of occurring headache after vaccination, hour		26.78 ± 6.93
Form of headache after vaccination (%)	Early (≤ 24 h)	109 (83.2)
	24 to 72 h)	10 (7.6)
	72 h to 7 days	8 (6.1)
	> 72 h	4 (3.1)
The mean duration of headache (hour)		4.22 ± 1.26
Pattern of post-vaccination headache (%)	Pressing	97 (74.0)
	Pulsatile	22 (16.8)
	Neurologic type	12 (9.2)
Location of post-vaccination headache (%)	Frontal	14 (10.7)
	Temporal	13 (9.9)
	Occipital	4 (3.1)
	Parietal	7 (5.3)
	Diffuse	43 (32.8)
	Neck	1 (0.7)
	Mixed	49 (37.5)
Other symptoms along with headache (%)	Nausea	21 (9.2)
	Photophobia	6 (4.6)
	Sensitivity to sound	9 (6.9)
Medication used for headache relief (%)	Acetaminophen	65 (49.6)
	Ibuprofen	17 (12.9)
	Naproxen	16 (12.2)
	Other analgesics	24 (18.3)

**Fig. 1:**
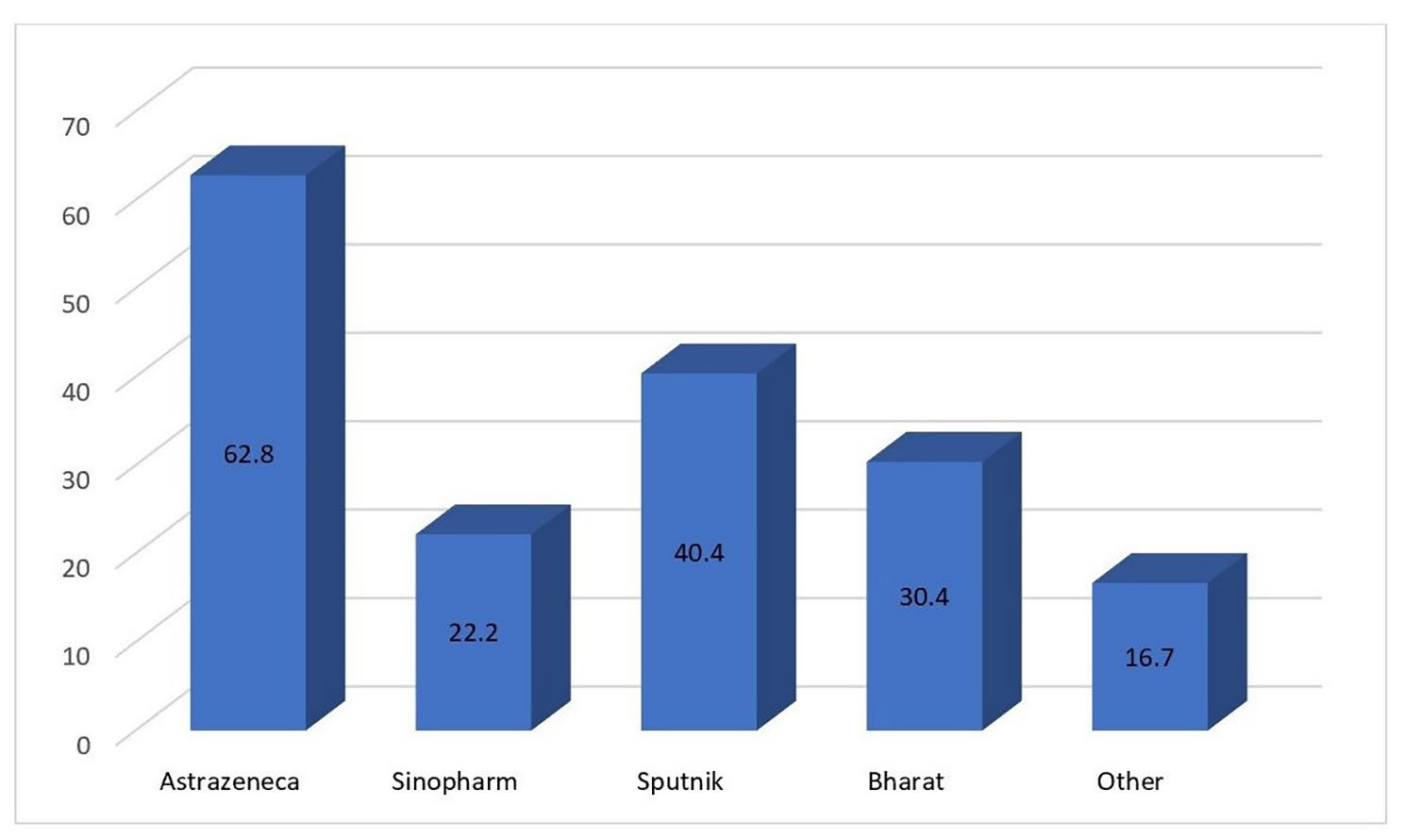
Prevalence of post-vaccine headache according to type of vaccine used (p = 0.001)

Figure 1 shows that the prevalence of post-vaccination headache was significantly different according to the brand of vaccine administered. The prevalence of post-vaccination headache was highest for AstraZeneca (62.8%), followed by Sputnik V (40.4%) and Bharat (30.4%) (p < 0.001). Post-vaccine headache was found to be significantly higher in females than in males (43.6% versus 27.5%; p = 0.001). Table 3 reveals that, although the onset of headache (early or delayed) did not differ across vaccine brands, the pattern of headache did differ. Compression headache was reported more often by those vaccinated with AstraZeneca or Sputnik V. Pulsatile headaches were reported to occur most often following vaccination with Sinopharm.

**Table 3:** The quality of headache according to type of vaccines used (n = 131)

Characteristics	Astrazeneca	Sinopharm	Sputnik	Baharat	P value
Form of headache					0.984
Early	23 (85.2)	10 (83.3)	69 (82.1)	6 (85.7)	
Delayed	4 (14.8)	2 916.7)	15 (17.9)	1 (14.3)	
Pattern of headache					0.046
Pressing	20 (74.1)	8 (66.7)	63 (75.0)	4 (57.1)	
Pulsatile	1 (3.7)	4 (33.3)	13 (15.5)	2 (28.6)	
Neuralgic type	6 (22.2)	0 (0.0)	8 (9.5)	1 (14.3)	
Location of headache					0.814
Frontal	0 (0.0)	2 (16.7)	10 (12.0)	1 (14.3)	
Temporal	2 (7.4)	1 (8.3)	10 (12.0)	0 (0.0)	
Occipital	0 (0.0)	0 (0.0)	4 (4.8)	0 (0.0)	
Parietal	3 (11.1)	0 (0.0)	4 (4.8)	0 (0.0)	
Diffuse	10 (37.0)	5 (41.7)	23 (27.7)	5 (71.4)	
Neck	0 (0.0)	0 (0.0)	1 (1.2)	0 (0.0)	
Mixed	12 (44.4)	4 (33.3)	31 (37.3)	1 (14.3)	

Table 4 indicates that, in multivariate logistic regression analysis, the brand of vaccine (OR = 1.328; p = 0.040), female gender (OR = 1.934; p = 0.017) and COVID-19 severity (OR = 3.541; p = 0.001) were the main determinants for prediction of post-vaccination headache. It was noted that a history of headache before vaccination was not significantly associated with the occurrence of post-vaccination headache.

**Table 4:** The effect of type of vaccine on post-vaccination headache adjusted for gender, history of headache, and COVID-19 severity

Factor	B	S.E.	Sig.	Exp(B)	95.0% C.I.for EXP(B)	
					Lower	Upper
Type of vaccine	0.283	0.138	0.040	1.328	1.013	1.740
Male gender	-0.660	0.277	0.017	0.517	0.301	0.889
History of headache	-0.240	0.127	0.059	0.787	0.613	1.009
COVID-19 severity	1.264	0.671	0.001	3.541	1.668	5.642

Hosmer-Lemeshow Goodness of Fit: Chi-Square = 9.428, p = 0.307

## Discussion

Recent studies have reported on the occurrence of headache after inoculation with COVID-19 vaccines; however, the present study is the first to evaluate this event in individuals who have been vaccinated after initial infection by and recovery from COVID-19 and who experienced post-vaccination headaches. It should be noted that, post-vaccination, there were no signs of re-infection among participants.

**Fig. 2:**
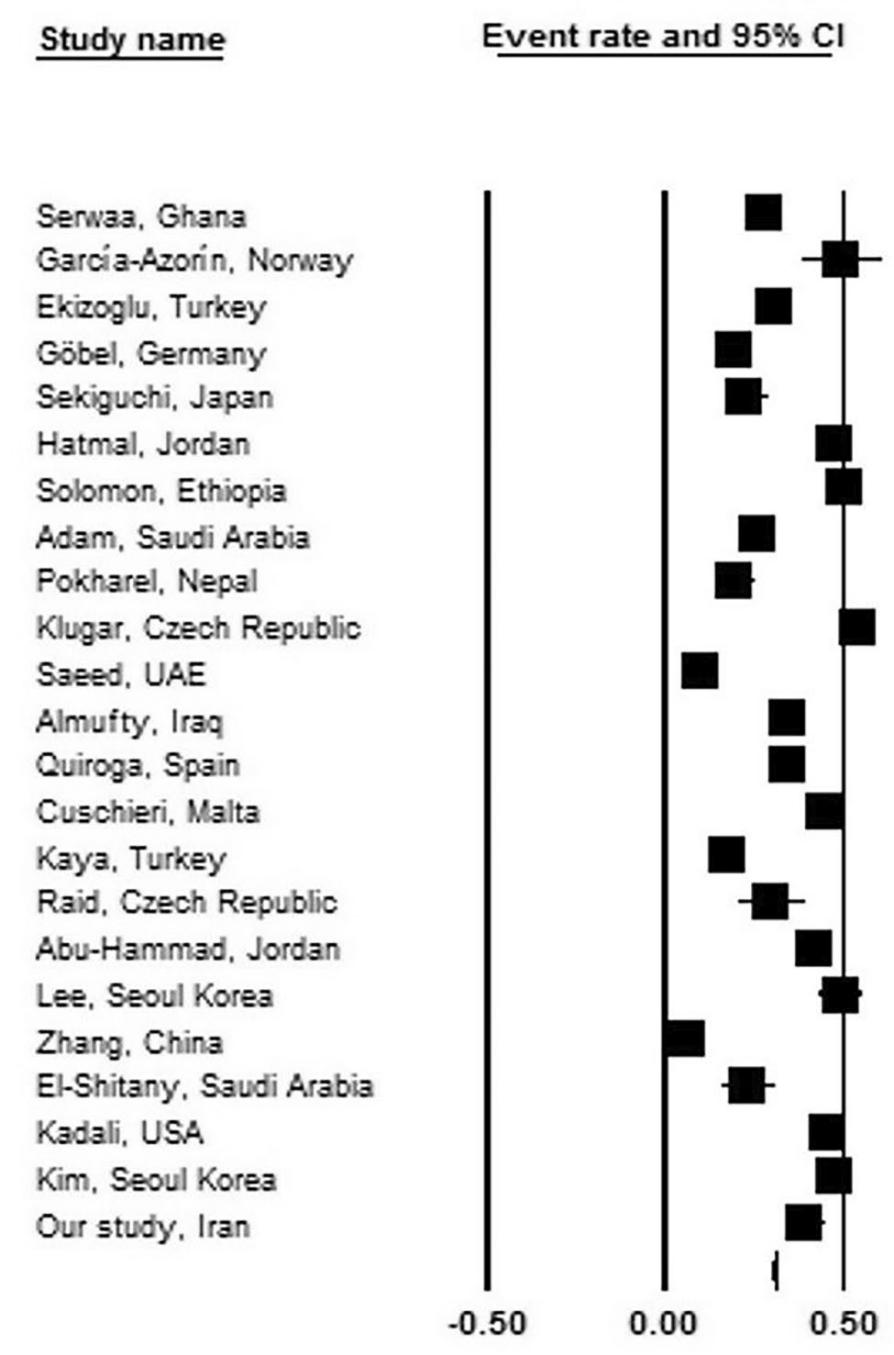
Prevalence of post-vaccine headache among total population in different studies

**Fig. 3:**
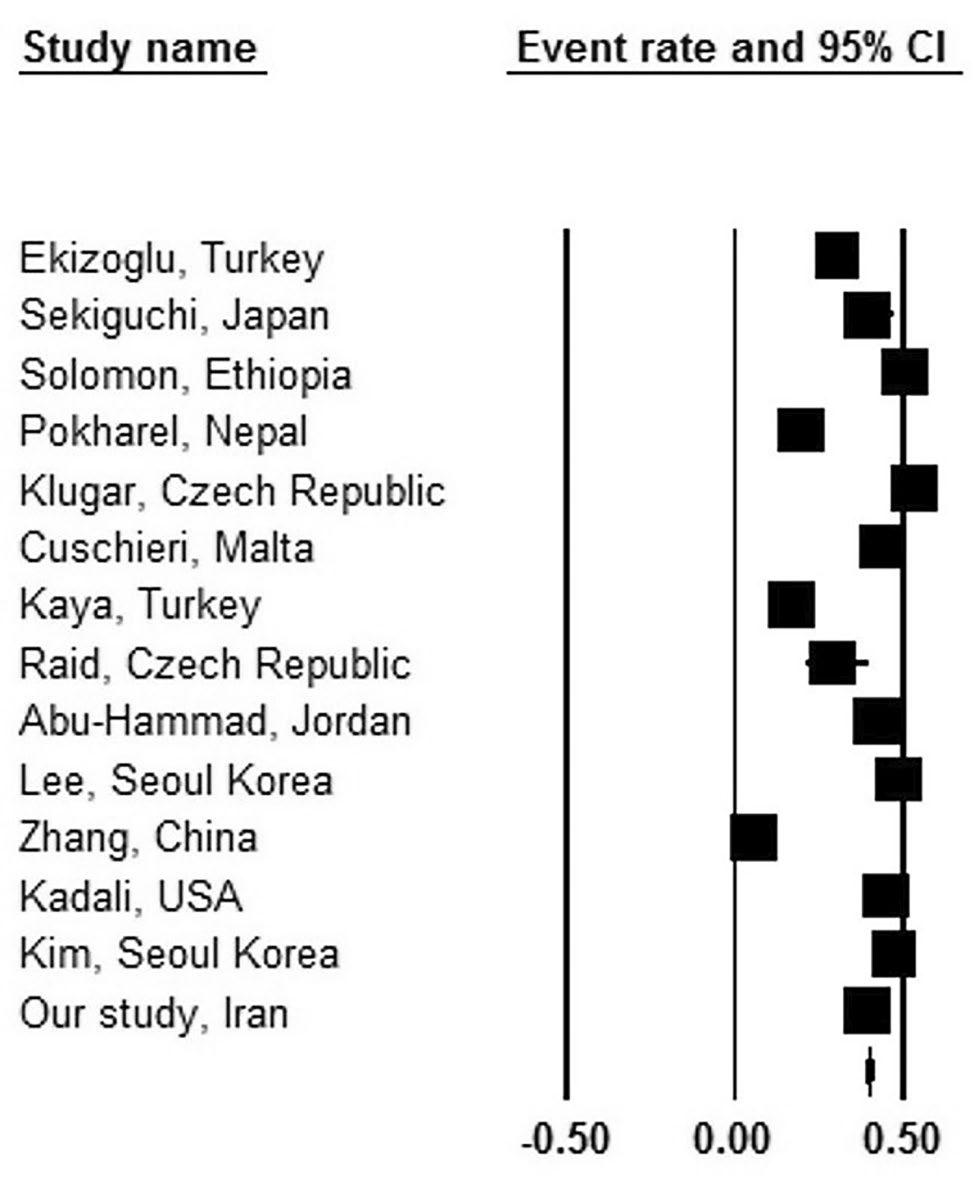
Prevalence of post-vaccine headache among healthcare workers in different studies

The predominant finding of the present study has been that about one-third of the vaccinated individuals in the study group reported various types of post-vaccination headache. A review of the literature (Table 5) showed that the incidence of post-vaccination headache ranged from 19.5 to 49.4% regardless of the type of vaccine used or the target population (general population or healthcare workers). In a meta-analysis of these studies, we found an overall prevalence of 31.2% (95% CI: 25.3–37.9%) for headache, with a prevalence of 34.6% (95% CI: 27.4–42.5%) among healthcare workers, but with considerable heterogeneity across the studies (I^2^ = 99.037 (Figs. 2) and 98.343 (Fig. 3), respectively; p < 0.001) [12–33]. These divergent results could relate to the different brands of vaccine used as well as differences in the study populations. It could be concluded that about one-third of individuals who have been vaccinated against COVID-19 experienced various degrees of headache, with a slightly higher incidence rate among healthcare personnel.

**Table 5:** Reviewing the studies on post-vaccination headache

Author, Country	Type of study	Number of population	Targeted population	Type of vaccine	Prevalence of headache
Serwaa, Ghana [13]	Cross-sectional	654	Personnel	AstraZeneca	27.3% 178
García-Azorín, Norway [14]	Cross-sectional	77	General	Non-replicant adenovirus vector-based vaccines	49.4% 38
Ekizoglu, Turkey [15]	Cross-sectional	1819	Personnel	CoronaVac (Pfizer)	30.6% 556
Göbel, Germany [16]	Cohort	12,000	General	ChAdOx1 nCoV-19	19.5% 2340
Sekiguchi, Japan [17]	Cross-sectional	171	Personnel	Pfizer	39.7% 68
Hatmal, Jordan [18]	Cross-sectional	2213	General	Sinopharm, AstraZeneca, Pfizer-BioNTech	46.9% 1038
Solomon, Ethiopia [18]	Cross-sectional	672	Personnel	AstraZeneca	50.2% 337
Adam, Saudi Arabia [19]	Cross-sectional	330	General	Pfizer, AstraZeneca	24.2% 86
Pokharel, Nepal, [20]	Cross-sectional	220	Personnel	Covishield	19.5% 43
Klugar, Czech Republic [21]	Cross-sectional	599	Personnel	Pfizer, AstraZeneca	53.6% 321
Saeed, UAE [22]	Cross-sectional	1102	General	Sinopharm	10.0% 110
Almufty, Iraq [23]	Cross-sectional	1012	General	Pfizer, AstraZeneca, Sinopharm	34.0% 344
Quiroga, Spain [24]	Cross-sectional	708	General	Pfizer	34.0% 240
Cuschieri, Malta [25]	Cross-sectional	1480	Personnel	Pfizer	44.2% 655
Kaya, Turkey [26]	Cohort	329	Personnel	Pfizer	16.8%, 56
Raid, Czech Republic [27]	Cross-sectional	92	Personnel	AstraZeneca	29.3%, 27
Abu-Hammad, Jordan [28]	Cross-sectional	409	Personnel	Pfizer, AstraZeneca, Sinopharm	42.0% 172
Lee, Seoul Korea [29]	Cross-sectional	265	Personnel	Pfizer	48.7% 129
Zhang, China [30]	Cross-sectional	1526	Personnel	Pfizer	6.0% 92
El-Shitany, Saudi Arabia [31]	Cross-sectional	124	General	Pfizer	22.5% 28
Kadali, USA [32]	Cross-sectional	1245	Personnel	Pfizer	45.4% 565
Kim, Seoul Korea [33]	Cross-sectional	1403	Personnel	Pfizer, AstraZeneca	47.4% 665
Our study, Iran	Cross-sectional	334	Personnel	AstraZeneca, Sinopharm Sputnik v Bharat, Co Iran barekat	39.2% 131

More interestingly, most headaches occurred within the first 24 h after vaccination (83.2%) with the mean time between vaccination and headache onset to be 26.78 ± 6.93 h. As indicated by Göbel et al. [34], the latency between vaccination against COVID-19 and the occurrence of headache was on average 18.0 ± 27.0 h. More than half of their participants perceived the headache in less than 10 h and 80% within 24 h after vaccination, which is similar to our findings. Koji Sekiguchi et al. [35] also reported that the median onset of headache after the first and second vaccine doses were 10 and 12 h, respectively, and mean duration of headache was 4.5 and 8.0 h, respectively. In that study, the mean time to onset of headache after vaccination was 4.22 ± 1.26 h. In 50% of their participants, the headache duration was less than 6 h and in 80% was less than 22 h. Göbel et al. [34] reported a mean headache duration of 14.2 + 21.4 h.

About one-third of participants reported generalized headache. Göbel et al. [34] reported bilateral headache in 73.1% of their subjects, with the most prominent zones being the forehead (38.0%) followed by the temple (32.2%). Sekiguchi et al. [35] reported the rate of bilateral headache in healthy controls having no history of headache, and history of migraine and non-migraine headaches as being 78.8%, 62.5% and 75.9%, respectively. The participants in the present study primarily reported compression-type headaches. Göbel et al. [34] reported compression headache and dull pain in 49.2% and 40.7% of participants, respectively. Ekizoglu et al. [15] reported throbbing headaches in 40.1% of participants and compression headache in 30.4%.

Another important finding was the occurrence of post-vaccination headache as being potentially influenced by the factors of the female gender and severity of the initial COVID-19 infection. Research released by the CDC on the safety of COVID-19 vaccinations indicated post-vaccination side-effects occurred among 79.1% of women but only in 61.2% of men [36]. As migraine and tension headaches are more prevalent in women than in men [37, 38], such a difference may affect the likelihood of post-vaccination headache among women compared to men.

The current study found a significant difference in the prevalence of headache according to vaccine brand used among different countries. As shown, the highest rate of headache was after AstraZeneca vaccination, followed by Sputnik V; however, the literature reviewed (Table 5) did not differentiate between vaccines in relation to post-vaccination headache. For example, the rate of post-vaccination headache following vaccination by Pfizer-BioNTech ranged from 6.0 to 48.7%. Additionally, information about the incidence of side-effects of brands such as Sinopharm and Sputnik V vaccines has been limited.

There is no documented and comprehensive explanation of the pathomechanisms of headache following vaccination against COVID-19. Some believe that such a headache may originate from the spike protein of the virus used to produce the vaccine [39]. Others have speculated that the immune response triggered by such proteins plays a significant role [40]. This means that flaring pro-inflammatory cascades and secretion of cytokines and prostaglandins may be responsible for vaccination-related headache and other concurrent symptoms [41, 41]. It should be noted that the technologies and materials used for creating the vaccines could play a role in post-vaccination headache. This should be evaluated in further studies.

One limitation of the study was that some of the most commonly used brands globally, such as Pfizer, were not widely available in Iran; thus was not possible to evaluate the post-vaccination headache for these brands. Additionally, the pattern of headache among the healthcare workers as participants was not evaluated during the first exposure to COVID-19.

## Conclusion

The current study examined the incidence of post-vaccination headache among healthcare workers who were vaccinated against COVID-19 after recovering from a previous bout of the virus. Different brands of vaccine were examined and it was found that 39.2% of participants experienced post-vaccination headache. This incidence was greater among females than males as well as those who had experienced more severe cases of COVID-19 before vaccination. Among the brands used in our population, the highest rate of post-vaccination headache was for the AstraZeneca, followed by Sputnik V. Considering that COVID-19 will continue to infect the global population in the future, vaccination, as well as identification and classification of post-vaccination headache, can improve appropriate management of the virus. The differentiation of such headaches from other post-vaccination side-effects, such as cerebrovascular thrombotic events, can be vital to the targeted management of these events.

## Data Availability

Data available on request due to privacy/ethical restrictions.
